# Workers’ Health Risk Behaviors by State, Demographic Characteristics, and Health Insurance Status

**Published:** 2010-12-15

**Authors:** Yi Huang, Peggy A. Hannon, Barbara Williams, Jeffrey R. Harris

**Affiliations:** University of Washington, Seattle, Washington; University of Washington; University of Washington, Seattle, Washington; University of Washington, Seattle, Washington

## Abstract

**Introduction:**

Employers often lack data about their workers' health risk behaviors. We analyzed state-level prevalence data among workers for 4 common health risk behaviors: obesity, physical inactivity, smoking, and missed influenza vaccination (among workers older than 50 years).

**Methods:**

We analyzed 2007 and 2008 Behavioral Risk Factor Surveillance System data, restricting the sample to employed respondents aged 18 to 64 years. We stratified health risk behavior prevalence by annual household income, educational attainment, health insurance status, and race/ethnicity.

**Results:**

For all 4 health risk behaviors, we found significant differences across states and significant disparities related to social determinants of health — income, education, and race/ethnicity. Among uninsured workers, prevalence of smoking was high and influenza vaccinations were lacking.

**Conclusion:**

In this national survey study, we found that workers' health risk behaviors vary substantially by state and by workers' socioeconomic status, insurance status, and race/ethnicity. Employers and workplace health promotion practitioners can use the prevalence tables presented in this article to inform their workplace health promotion programs.

## Introduction

Health risk behaviors are common among workers, are strongly related to chronic illness and death, increase health care costs, and reduce productivity (1). One key to a successful workplace health promotion program is to measure workers' baseline health needs and use the data to inform the program (2,3). However, most employers do not have access to data about their workers' health behaviors. Many midsized and small employers lack the resources to conduct health risk appraisals (HRAs). In addition, employer-run HRAs often have low response rates and overrepresent healthy workers (4).

Readily available data about risk behaviors could help employers plan and evaluate their workplace health promotion programs. Obesity, physical inactivity, and tobacco use are 3 of the most common lifestyle health risk behaviors in the United States (5,6) and cause approximately one-third of all deaths (7). Influenza vaccination is also of interest to employers because influenza leads to lost productivity and can trigger severe pulmonary and cardiovascular diseases. Vaccination reduces the incidence of influenza and can save employers money in a short time frame (1 year or less) (8).

The objective of this study was to provide employers and other workplace health promotion practitioners with state-specific data for these 4 health risk behaviors (obesity, physical inactivity, smoking, and no influenza vaccination [among workers older than 50 years]) among workers. We stratified the behaviors by insurance status and social determinants of health: annual household income, educational attainment, and race/ethnicity. To meet this objective, we show the prevalence of each health risk behavior by state and workers' characteristics, using data from the 2007 and 2008 Behavioral Risk Factor Surveillance System (BRFSS), the most recent data available.

## Methods

### Design

We conducted a cross-sectional study by using BRFSS data collected in 2007 and 2008. With assistance from the Centers for Disease Control and Prevention (CDC), state health departments conduct BRFSS surveys among US resident civilian, noninstitutionalized adults aged 18 years or older in all 50 states, the District of Columbia, and US territories (9).

Using a multistage cluster design, BRFSS selects state-specific probability samples of households to produce a nationally representative sample (5). After calling a selected home telephone number, the interviewer randomly chooses 1 adult in that household to complete the telephone interview. BRFSS data are weighted by race/ethnicity, age, and sex distributions found in each state, along with the respondent's probability of selection.

### Sample

The median cooperation rate, or the proportion of all respondents interviewed from all eligible units in which a respondent was selected and contacted, was 72.1% in 2007 and 75.0% in 2008 (10,11). Our study population included employed adults aged 18 to 64 years in 50 states and the District of Columbia. We considered adults *employed* if they were employed for wages or self-employed. We excluded adults older than 64 years because Medicare is available for most of this group.

### Measures

The BRFSS questionnaire has 3 parts: core questions, optional modules, and state-added questions. All states must ask core questions every year or every other year. States may also choose optional modules or add their own questions to meet their specific data needs. Both English- and Spanish-language versions of the survey are provided to each state.

In this article, all data are from the core questions used in every state. The health risk behaviors are lifestyle behaviors (obesity, physical inactivity, and smoking) and no influenza vaccination in the past year. Obesity is defined as having a body mass index of at least 30 kg/m^2^ (12). Physical inactivity is defined as not meeting the CDC physical activity guideline of at least 5 days per week for 30 minutes per day of moderate-intensity activity or at least 3 days per week for 20 minutes a day of vigorous-intensity activity (13,14). Tobacco use is defined as ever having smoked at least 100 cigarettes and currently smoking every day or some days. Workers aged 50 to 64 years who reported no influenza vaccination in the past 12 months (either by injection or nasal spray) were defined as not vaccinated. We restricted the influenza vaccination analysis to workers older than age 50 because CDC's Advisory Committee on Immunization Practices recommends influenza vaccination for those adults (15).

We analyzed workers' socioeconomic status (SES), race/ethnicity, health insurance status, and health risk behaviors. The SES measures are annual household income and educational attainment as reported in the BRFSS data. We used 2007 BRFSS data for the physical inactivity measure because these questions were not included in the 2008 survey. We used 2008 data for the rest of the measures.

### Analysis

We calculated national and state rates for workers stratified by 1) annual household income (<$35,000, $35,000-$74,999, ≥$75,000), 2) educational attainment (high school graduate or less, some college, college graduate), 3) health insurance (any, none), and 4) race/ethnicity (African American, American Indian/Alaska Native, Asian/Hawaiian/Pacific Islander, Hispanic, and white). We identified the national prevalence of each health risk behavior among workers, the range across states, and the range across states for characteristics associated with the highest risk behavior prevalence nationally.

Our analysis took into account the survey design and weighted sampling probabilities of the data source and was performed by using Stata version 10.0 (StataCorp LP, College Station, Texas). All the statistical tests were 2-sided and significance was set at *P* < .05. We calculated 95% confidence intervals (CIs) for all prevalence rates (versions of the tables with CIs are available from the corresponding author on request). Because of the very small numbers of respondents in some categories, we restricted the prevalence estimates to the categories in which there were 50 or more respondents.

## Results

### Final sample

There were 430,912 respondents in the 2007 BRFSS, and 414,509 respondents in the 2008 BRFSS. When we restricted our data sample to employed respondents aged 18 to 64 years, 48.3% of the 2007 sample (physical inactivity) and 47.5% of the 2008 sample (obesity, smoking, and influenza vaccination) remained. For each of the analyses described below, we excluded respondents who were missing data for the health risk behavior under study; therefore, the number of subjects varies slightly across the analyses. We further excluded respondents who were missing data for SES, insurance status, or race/ethnicity from all analyses stratified by these characteristics (8.3% in 2007 and 8.0% in 2008 were missing 1 or more of these variables). Thus, of the respondents who met our employment and age criteria, we were able to include more than 85% in our analyses (range: 87.0% for physical activity to 91.8% for smoking).

### Obesity

In 2008, 27.0% of employed adults in the United States were obese (Table 1); obesity rates were lowest in Colorado (19.5%) and were highest in West Virginia (34.6%). Nationally, the highest obesity rates were reported by those with annual household incomes less than $35,000 (30.2%), those who did not graduate from college (30.5%), and African Americans (37.3%). Obesity rates among workers with these characteristics varied significantly across states, from 21.8% (95% CI, 18.3%-25.2%) in Colorado to 39.2% (95% CI, 35.0%-43.4%) in Mississippi for low-income workers; from 23.5% (95% CI, 21.0%-26.1%) in Massachusetts to 39.1% (95% CI, 33.1%-45.1%) in Tennessee among workers with a high school education or less; and from 17.9% (95% CI, 6.5%-29.4%) in Nevada to 49.9% (95% CI, 33.3%-66.4%) in Nebraska for African American workers.

### Physical inactivity

In 2007, 49.2% of employed adults did not meet physical activity recommendations (Table 2); physical inactivity rates were lowest in Alaska (37.2%) and highest in Louisiana (58.4%). Nationally, the highest physical inactivity rates were reported by workers with household incomes less than $35,000 (54.3%), high school education or less (52.5%), and Asians/Hawaiians/Pacific Islanders (63.1%). Physical inactivity rates for workers with these characteristics varied significantly across states, from 42.5% (95% CI, 37.8%-47.2%) in Montana to 68.7% (95% CI, 63.0%-74.3%) in Tennessee for low-income workers; from 36.1% (95% CI, 29.4%-42.8%) in Alaska to 61.0% (95% CI, 57.0%-65.1%) in Louisiana for workers with a high school education or less; and from 40.1% (95% CI, 22.1%-58.1%) in Pennsylvania to 70.2% (95% CI, 63.3%-77.1%) in California for Asian/Hawaiian/Pacific Islander workers.

### Smoking

In 2008, 19.2% of employed adults reported that they currently smoke cigarettes (Table 3); smoking rates were lowest in Utah (9.8%) and highest in Indiana (27.6%). Nationally, the highest smoking rates were reported by workers with household incomes less than $35,000 (28.9%), high school education or less (29.3%), no health insurance (32.5%), and American Indians/Alaska Natives (27.8%). Among workers with these characteristics, smoking rates varied significantly across states, from 15.3% (95% CI, 11.1%-19.5%) in Utah to 45.6% (95% CI, 38.4%-52.8%) in Indiana for low-income workers; from 17.6% (95% CI, 14.2%-21.0%) in Utah to 41.1% (95% CI, 35.7%-46.5%) in Indiana for workers with high school education or less; from 13.8% (95% CI, 9.1%-18.5%) in Utah to 54.9% (95% CI, 45.9%-63.9%) in Indiana for uninsured workers; and from 10.9% (95% CI, 2.3%-19.5%) in Arizona to 53.1% (95% CI, 32.6%-73.5%) in North Dakota for American Indian/Alaska Native workers.

### No influenza vaccination

In 2008, 59.3% of workers aged 50 to 64 years reported no influenza vaccination (Table 4); the lowest rate was in South Dakota (47.1%) and the highest was in Nevada (71.4%). Nationally, workers most likely to report no influenza vaccination had household income less than $35,000 (68.6%), high school education or less (66.3%), no health insurance (77.1%), and were Hispanic (67.1%). Among workers with these characteristics, rates of no influenza vaccination varied significantly across states, from 49.0% in Virginia (95% CI, 36.3%-61.7%) to 83.3% (95% CI, 77.1%-89.4%) in Nevada for low-income workers; from 51.6% (95% CI, 46.6%-56.6%) in South Dakota to 82.0% (95% CI, 75.5%-88.5%) in Nevada for workers with a high school education or less; from 59.5% (95% CI, 47.6%-71.4%) in Iowa to 90.2% (95% CI, 83.3%-97.1%) in Indiana for uninsured workers; and from 50.9% (95% CI, 34.7%-67.0%) in Hawaii to 84.3% (95% CI, 75.0%-93.6%) in Nevada for Hispanic workers.

## Discussion

The most effective workplace health promotion efforts are tailored to the risk behaviors and needs of the workers (2,3). However, for many employers, data describing their workers are unavailable or unrepresentative of their workforce (4,16). To address this need, we used BRFSS data, a very large, recent data set of employed adults in the United States, and calculated prevalence for 4 common health risk behaviors stratified by state and by the worker characteristics that employers routinely collect to describe their workforce.

In this national sample of employed adults aged 18 to 64 years, we found significant disparities related to SES and race/ethnicity for all 4 health risk behaviors and significant disparities by insurance status for smoking and influenza vaccination. We also found significant variations in health risk behaviors within and across states. Our findings both replicate and extend our prior study of employed workers' health risk behaviors, which found significant disparities by SES and race/ethnicity among insured workers (6). The findings make state-level data for workers available for the first time, include uninsured workers, and show that disparities are worse for the uninsured for influenza vaccination and tobacco use than for obesity and physical inactivity.

### Limitations

Our study and prevalence tables have several limitations. First, BRFSS includes only people who have home telephones and speak either English or Spanish. Second, all of the health risk behaviors are self-reported. These 2 limitations suggest that our results may underreport the prevalence of workers' health risk behaviors. Third, in many states, fewer than 50 members of some racial/ethnic groups were included in the sample, and we were not able to present health risk behavior rates in these cases. In other states, we were able to present health risk behavior rates for every racial/ethnic group, but some of the confidence intervals are wide because of small numbers in these groups. Fourth, our study was cross-sectional; our findings show associations between characteristics and health risk behaviors but not causation.

An important limitation of our study is that the prevalence tables are at the state rather than the local level. As such, they cannot provide employers with as accurate a view of their workers' health risk behaviors as they could achieve by surveying their workers. For many employers, acquiring health behavior data from their own workers is often not feasible. Finally, our findings do not address the time and financial challenges employers face in implementing workplace health promotion programs. However, our findings can serve employers by 1) providing data on the health risks of workers in their state with similar characteristics to those of their own workforce (comparable to the intent of county health-ranking systems that motivate policy makers to take action to improve health risks in their counties [17]) and 2) serving as a planning tool for an individual employer's health promotion efforts.

### Conclusion

To our knowledge, this is the first time that state-level BRFSS tables summarizing health risk behaviors of the US *employed* population have been made available. We found significant differences in workers' health behaviors across states and within states, depending on their SES, insurance status, and race/ethnicity. Employers, workplace health promotion professionals, insurers, and vendors can use these tables to inform workplace health promotion planning when data for a given employer's workers are not available.

## Figures and Tables

**Table 1:** Prevalence of Obesity^a^ by State Among Workers Aged 18 to 64 Years, 2008 Behavioral Risk Factor Surveillance System (BRFSS)

State	No. of Respondents^c^	Prevalence of Obesity^b^, %													

		Overall, %	Annual Household Income, $			Educational Attainment			Health Insurance Status		Race/Ethnicity				

			<35,000	35,000-<74,999	≥75,000	High School Graduate or Less	Some College	College Graduate	Any	None	White	African American	Asian/ Hawaiian/ Pacific Islander	American Indian/ Alaska Native	Hispanic
Alabama	2,561	32.3	37.2	36.4	26.7	35.0	35.9	26.7	32.9	29.4	30.3	39.1	—	—	—
Alaska	1,631	25.3	25.2	25.7	26.5	26.9	28.6	20.4	27.5	15.8	25.8	—	—	34.0	9.4
Arizona	2,214	25.7	26.2	32.1	23.2	29.6	24.8	23.1	25.3	27.5	22.7	—	—	49.5	31.5
Arkansas	2,369	30.8	34.1	33.9	25.9	33.7	31.0	27.3	31.1	30.1	30.4	37.0	—	—	30.8
California	5,258	24.5	27.5	26.5	21.3	28.9	27.0	18.5	24.1	26.5	23.2	33.3	7.5	—	29.3
Colorado	5,946	19.5	21.8	20.0	18.2	23.7	22.4	15.1	19.3	20.6	18.2	27.6	5.7	—	25.4
Connecticut	2,884	20.6	24.3	22.2	19.6	24.7	22.8	17.5	20.6	19.4	19.8	30.2	9.7	—	28.9
Delaware	1,934	29.0	36.4	32.0	24.8	33.4	34.8	22.7	28.2	39.0	25.7	47.6	—	—	46.8
District of Columbia	2,170	20.9	28.4	26.8	15.9	32.3	36.9	14.1	20.5	26.1	9.6	34.4	—	—	19.9
Florida	4,353	25.0	30.3	23.7	24.8	32.4	25.8	18.4	25.1	24.1	23.5	33.2	—	—	28.3
Georgia	2,650	27.6	32.3	28.6	24.8	32.0	34.7	20.6	27.4	29.1	24.2	39.1	—	—	27.5
Hawaii	3,466	23.2	25.0	23.9	21.6	27.4	25.2	18.6	23.4	20.2	19.0	—	17.4	—	29.2
Idaho	2,382	26.1	29.2	26.2	23.7	25.2	32.8	21.2	25.7	27.7	25.3	—	—	—	26.3
Illinois	2,494	27.9	33.3	29.8	24.4	31.4	32.9	22.2	26.5	36.0	25.6	38.9	15.8	—	33.8
Indiana	2,299	26.7	26.0	30.0	25.8	24.9	31.3	25.6	28.1	20.4	27.5	36.5	—	—	15.0
Iowa	3,069	27.2	35.1	28.4	23.2	29.9	30.4	21.8	27.1	28.5	26.9	—	—	—	34.6
Kansas	4,352	29.3	31.1	32.5	26.6	31.4	31.7	26.1	29.8	24.4	28.8	48.5	—	—	33.2
Kentucky	3,225	31.0	36.7	32.2	26.4	32.8	32.3	28.1	30.6	31.5	30.0	48.5	—	—	—
Louisiana	2,738	29.4	33.3	34.8	24.6	32.7	31.4	24.8	29.4	29.0	26.6	35.3	—	—	35.9
Maine	3,267	26.4	27.8	29.7	22.5	30.7	30.8	20.2	26.2	27.9	26.5	—	—	—	—
Maryland	4,787	26.3	29.6	27.8	25.3	29.7	31.8	22.1	26.3	26.4	23.4	35.2	18.8	—	22.9
Massachusetts	10,188	21.6	23.3	23.8	20.3	23.5	25.9	18.6	21.7	20.2	21.6	28.2	4.5	—	26.0
Michigan	3,918	28.9	31.9	31.9	25.6	30.2	33.9	24.0	28.8	29.4	28.3	43.6	—	—	19.8
Minnesota	2,299	25.2	29.0	25.9	24.1	27.1	27.9	22.4	26.0	16.2	25.6	23.3	—	—	—
Mississippi	3,181	34.5	39.2	35.6	28.6	36.3	36.1	30.9	34.2	35.2	31.3	41.4	—	—	42.0
Missouri	2,314	30.6	31.3	32.5	27.7	29.4	36.0	27.9	30.6	31.4	29.9	34.9	—	—	—
Montana	3,204	24.3	28.9	23.0	21.4	26.6	28.1	19.8	23.7	26.6	23.5	-	—	43.3	21.9
Nebraska	8,285	28.0	29.2	32.1	26.1	28.8	33.2	23.4	28.3	26.9	27.6	49.9	—	—	25.4
Nevada	2,244	26.5	30.3	27.2	23.5	30.5	25.3	22.6	26.3	28.0	23.9	17.9	22.4	—	33.5
New Hampshire	3,460	24.5	27.2	26.7	22.6	27.2	29.3	20.7	24.6	24.8	24.6	—	—	—	—
New Jersey	5,706	23.4	25.8	27.2	21.4	28.8	26.5	18.4	23.5	22.8	23.3	34.1	8.4	—	24.3
New Mexico	2,880	26.9	29.1	27.8	23.1	30.5	30.8	20.3	26.9	26.5	21.3	—	—	35.1	31.8
New York	3,634	25.3	26.8	29.6	22.8	28.6	30.3	20.6	25.2	26.8	24.1	34.2	7.4	—	30.2
North Carolina	7,070	30.8	34.0	32.5	27.9	34.2	35.2	23.9	29.5	36.4	29.1	41.4	5.7	37.6	26.7
North Dakota	2,643	28.8	30.6	30.6	26.7	32.3	28.9	25.8	28.9	25.8	28.6	—	—	47.6	—
Ohio	5,740	29.8	33.7	34.1	26.1	32.4	32.8	25.7	30.2	26.7	29.4	37.0	8.4	—	38.5
Oklahoma	3,317	32.3	32.2	34.8	30.8	31.6	35.7	30.5	33.1	29.0	31.2	41.0	—	39.3	33.0
Oregon	2,147	24.8	27.2	26.6	22.3	26.5	28.9	20.6	25.3	21.9	25.4	—	—	—	17.7
Pennsylvania	5,658	29.9	36.0	30.6	25.8	34.4	33.4	23.2	29.3	34.9	29.1	42.1	9.6	—	32.0
Rhode Island	2,237	22.9	29.5	23.5	21.4	27.3	24.2	19.4	22.8	23.8	21.6	30.3	—	—	27.8
South Carolina	4,217	30.9	39.0	31.7	24.9	35.3	33.4	24.7	29.9	36.2	26.6	43.4	—	—	34.0
South Dakota	3,491	29.1	34.4	30.3	24.4	32.6	30.7	24.7	28.9	30.8	28.5	—	—	41.1	—
Tennessee	1,896	32.3	33.9	34.5	25.6	39.1	33.3	23.4	32.5	31.4	29.9	38.4	—	—	—
Texas	4,525	29.1	30.1	33.1	27.2	30.0	35.1	24.4	29.8	27.3	28.2	37.7	3.7	—	31.7
Utah	2,838	24.0	28.2	24.6	23.3	25.9	23.1	23.0	24.0	24.4	24.4	—	—	—	20.8
Vermont	3,715	22.6	23.2	25.2	19.2	27.5	25.7	17.2	22.5	23.7	22.7	—	—	—	—
Virginia	2,389	26.1	24.3	26.8	25.4	30.4	27.9	23.0	26.9	18.5	27.3	33.7	—	—	17.7
Washington	10,222	26.1	26.6	28.4	24.7	29.7	30.2	20.8	26.4	23.8	26.5	28.3	15.0	36.8	25.5
West Virginia	1,729	34.6	34.5	35.7	32.5	37.1	36.5	29.4	34.6	34.8	34.4	—	—	—	—
Wisconsin	3,700	27.2	32.6	28.2	21.7	29.3	30.4	22.8	26.7	31.0	26.1	42.0	—	43.1	29.0
Wyoming	4,159	26.3	26.6	27.7	26.3	27.7	28.5	22.6	27.3	21.7	25.9	—	—	43.1	25.9
**United States**	189,055	27.0	30.2	29.3	24.1	30.5	30.5	21.9	26.9	27.6	26.1	37.3	9.1	32.5	29.3

a Obesity is defined as having a body mass index ≥30 kg/m^2^.

b The total number of employed respondents in the 2008 BRFSS data stratified by 50 states and Washington DC (excluding respondents missing obesity data).

c We restricted the prevalence estimates to the categories in which there were 50 or more respondents; blank cells indicate fewer than 50 respondents in this category. Confidence intervals are available from the authors on request.

**Table 2:** Prevalence of Physical Inactivity^a^ by State Among Workers Aged 18 to 64 Years, 2007 Behavioral Risk Factor Surveillance System (BRFSS)

State	No. of Respondents^c^	Prevalence of Physical Inactivity^b^, %													

		Overall, %	Annual Household Income, $			Educational Attainment			Health Insurance Status		Race/Ethnicity				

			<35,000	35,000-<74,999	≥75,000	High School Graduate or Less	Some College	College Graduate	Any	None	White	African American	Asian/ Hawaiian/ Pacific Islander	American Indian/ Alaska Native	Hispanic
Alabama	2,843	54.4	59.3	53.5	49.1	55.8	56.3	50.8	53.9	57.4	53.1	59.5	—	—	—
Alaska	1,475	37.2	43.1	38.1	31.6	36.1	38.7	37.0	36.2	43.2	36.4	—	—	47.3	-
Arizona	1,891	45.0	56.1	46.9	36.3	54.5	42.4	38.9	44.0	49.4	41.5	—	—	32.0	55.7
Arkansas	2,348	51.5	51.0	53.2	49.4	52.7	52.0	49.7	51.6	52.2	50.7	56.8	—	—	57.3
California	2,711	51.0	57.9	53.5	43.8	55.3	52.8	46.1	51.0	50.9	42.5	55.4	70.2	—	56.1
Colorado	6,245	43.8	51.5	45.4	37.0	50.2	45.6	38.5	42.4	50.6	41.4	52.8	40.4	—	52.4
Connecticut	3,537	45.9	55.4	43.8	44.6	47.2	46.0	45.2	45.6	48.7	43.9	55.6	68.9	—	53.7
Delaware	1,989	49.2	55.5	48.4	48.5	52.4	44.2	50.0	49.6	45.2	47.3	55.7	—	—	62.0
District of Columbia	2,005	41.3	54.7	44.8	35.9	58.4	42.0	36.7	41.0	45.6	31.6	47.6	45.1	—	57.5
Florida	16,435	50.8	56.3	50.4	46.7	56.1	49.0	47.7	49.8	55.0	48.5	53.7	46.1	47.2	58.4
Georgia	3,394	49.6	49.9	49.7	50.9	50.6	48.6	49.8	50.1	46.6	48.7	55.1	—	—	37.1
Hawaii	3,681	48.7	57.6	48.5	45.1	51.5	51.0	44.5	48.9	46.0	39.9	—	58.0	—	51.9
Idaho	2,566	43.2	49.2	42.2	39.3	45.3	42.8	40.9	42.4	46.1	42.8	—	—	—	50.9
Illinois	2,586	49.2	54.4	52.4	43.0	53.4	49.3	46.6	48.7	52.9	45.8	52.9	62.2	—	58.2
Indiana	2,809	48.8	52.9	50.1	43.2	51.6	48.4	46.0	49.3	46.5	48.6	45.4	—	—	53.7
Iowa	2,822	49.5	49.6	51.6	44.8	50.1	48.2	50.0	50.6	42.2	49.2	—	—	—	60.6
Kansas	4,384	48.7	54.4	48.6	44.6	52.9	46.1	47.5	48.4	52.5	48.3	48.7	—	—	55.4
Kentucky	2,398	50.0	51.3	52.4	44.5	50.9	49.9	48.6	50.3	48.3	51.1	41.3	—	—	—
Louisiana	3,013	58.4	62.9	59.1	55.3	61.0	60.5	54.4	57.4	62.1	58.9	55.3	—	—	71.1
Maine	3,391	41.1	44.5	41.7	38.6	41.2	44.6	38.6	42.8	30.9	40.9	-	—	—	—
Maryland	4,315	50.3	59.7	51.8	46.8	53.6	54.0	46.9	50.0	52.3	46.6	55.7	55.0	—	62.9
Massachusetts	9,867	46.3	55.8	47.1	43.7	52.4	46.8	43.5	45.9	52.1	44.0	54.0	61.2	—	56.4
Michigan	3,290	47.5	48.2	49.9	45.0	46.9	49.2	46.7	48.3	41.8	46.8	53.4	—	—	28.1
Minnesota	2,615	49.1	54.9	49.0	45.9	55.9	49.6	44.7	48.4	55.9	48.4	55.1	—	—	—
Mississippi	3,296	57.3	57.6	58.7	52.8	57.3	60.1	54.8	56.4	60.9	55.5	61.3	—	—	51.5
Missouri	2,448	50.3	47.7	47.6	51.6	48.6	53.0	49.8	51.3	43.5	48.8	64.7	—	—	—
Montana	2,895	39.5	42.5	39.8	34.8	38.2	42.2	38.6	38.1	45.3	39.4	—	—	48.4	29.9
Nebraska	5,540	46.2	52.0	45.2	41.7	50.9	41.3	46.0	46.0	47.6	45.7	—	—	—	51.4
Nevada	2,040	47.6	51.1	48.0	44.2	51.2	45.4	45.6	47.2	49.2	46.3	—	—	—	52.8
New Hampshire	2,982	45.1	47.4	48.8	41.7	49.3	43.6	43.2	45.5	41.9	44.8	—	—	—	44.7
New Jersey	3,153	49.8	58.7	50.6	46.2	53.9	51.5	46.7	48.0	61.8	45.0	54.8	69.9	—	57.3
New Mexico	3,093	44.4	50.3	39.4	40.5	51.5	41.3	39.4	42.7	49.9	40.6	—	—	44.2	47.7
New York	3,107	48.0	51.3	49.3	44.8	51.9	45.6	46.6	47.4	52.0	44.1	51.6	61.3	—	59.2
North Carolina	6,560	53.5	58.2	53.8	47.9	59.7	52.2	48.4	53.0	56.2	50.7	58.1	54.2	50.4	70.3
North Dakota	2,579	45.6	45.2	46.0	42.1	47.4	45.4	44.2	45.4	45.1	45.9	—	—	46.4	—
Ohio	5,013	48.1	51.3	48.0	45.7	48.3	49.8	46.7	48.2	46.5	47.5	52.4	—	—	69.0
Oklahoma	3,091	52.0	55.0	52.3	47.6	54.4	53.8	47.7	52.2	51.1	51.3	44.2	—	54.5	57.0
Oregon	2,237	43.0	47.1	44.4	38.0	47.8	43.7	39.0	42.4	45.5	42.3	—	—	—	52.0
Pennsylvania	5,735	45.7	47.6	47.4	43.0	48.7	46.1	42.9	46.3	40.0	46.6	48.1	40.1	—	39.7
Rhode Island	2,098	47.1	55.5	49.3	43.2	52.5	49.3	42.2	47.4	45.4	45.8	50.5	—	—	58.5
South Carolina	4,586	51.0	55.6	49.5	48.6	53.0	51.2	48.7	50.3	54.3	49.7	56.8	—	—	41.2
South Dakota	3,498	49.8	54.6	52.0	41.6	56.8	49.1	43.9	49.7	50.8	49.6	—	—	54.1	—
Tennessee	2,030	57.9	68.7	55.5	47.2	60.8	58.4	53.2	56.8	65.7	58.5	60.6	—	—	—
Texas	7,287	52.0	55.6	52.4	48.2	54.0	52.2	49.6	52.1	52.0	49.0	53.8	66.8	43.0	54.7
Utah	2,733	43.7	50.2	43.2	37.8	49.6	42.2	38.9	43.5	44.3	42.3	—	—	—	49.2
Vermont	3,724	40.3	45.3	40.4	37.9	46.9	42.9	34.1	41.3	33.9	40.0	—	—	—	55.7
Virginia	2,893	49.3	54.5	46.7	47.2	51.9	50.4	47.5	48.8	53.7	47.9	51.2	62.4	—	58.7
Washington	11,957	45.4	49.9	45.8	43.1	46.9	45.7	44.1	45.1	47.8	44.4	32.3	50.8	43.3	58.7
West Virginia	1,888	50.4	49.3	51.6	49.7	48.8	50.3	53.2	52.1	42.1	50.7	—	—	—	—
Wisconsin	3,867	42.7	46.1	43.8	39.1	41.7	46.5	40.4	42.7	42.5	42.8	44.9	—	—	31.2
Wyoming	3,229	41.5	44.7	41.3	39.0	42.9	43.7	37.5	41.9	40.6	40.9	—	—	—	49.0
**United States**	196,169	49.2	54.3	49.8	45.0	52.5	49.5	46.3	48.9	51.0	46.8	54.3	63.1	44.3	55.6

a Physical inactivity is defined as not meeting the Centers for Disease Control and Prevention physical activity guideline of at least 5 days per week for 30 minutes a day of moderate-intensity activity or at least 3 days per week for 20 minutes a day of vigorous-intensity activity.

b We restricted the prevalence estimates to the categories in which there were 50 or more respondents; blank cells indicate fewer than 50 respondents in this category. Confidence intervals are available from the authors on request.

c The total number of employed respondents in the 2007 BRFSS data stratified by 50 states and Washington. DC (excluding respondents missing physical inactivity data).

**Table 3:** Prevalence of Smoking^a^ by State Among Workers Aged 18 to 64 Years, 2008 Behavioral Risk Factor Surveillance System (BRFSS)

State	No. of Respondents^c^	Prevalence of Smoking b, %													

		Overall, %	Annual Household Income, $			Educational Attainment			Health Insurance Status		Race/Ethnicity				

			<35,000	35,000-<74,999	≥75,000	High School Graduate or Less	Some College	College Graduate	Any	None	White	African American	Asian/ Hawaiian/ Pacific Islander	American Indian/ Alaska Native	Hispanic
Alabama	2,659	22.9	29.7	22.3	13.5	32.9	20.1	13.6	20.1	38.7	23.3	22.6	—	—	—
Alaska	1,658	20.1	32.8	17.2	14.4	33.8	16.9	7.9	17.8	30.5	16.7	—	—	35.1	15.0
Arizona	2,330	17.4	25.8	20.4	9.7	28.1	18.8	6.4	14.8	29.6	17.8	—	—	10.9	18.0
Arkansas	2,474	23.0	36.9	21.6	11.4	34.0	24.3	8.7	19.4	38.9	22.0	26.1	—	—	29.5
California	5,391	14.9	20.6	15.9	9.7	19.3	20.5	6.9	13.4	21.2	15.3	22.4	6.9	—	14.9
Colorado	6,157	17.9	28.3	21.8	10.2	29.6	21.6	7.6	15.7	29.5	16.5	21.4	12.9	—	21.7
Connecticut	3,006	17.5	29.3	22.0	12.2	28.8	23.3	9.6	16.2	31.1	17.4	16.5	12.0	—	20.0
Delaware	2,015	19.4	35.6	23.3	11.9	35.0	19.5	9.4	18.4	32.0	19.9	17.3	—	—	17.9
District of Columbia	2,241	14.6	24.5	19.7	8.5	27.5	22.2	9.0	13.3	28.6	10.3	20.7	—	—	11.7
Florida	4,515	19.3	29.2	18.3	11.7	25.5	23.6	11.1	16.3	32.9	22.2	9.8	—	—	17.1
Georgia	2,719	19.4	27.3	21.1	15.0	31.3	20.3	10.5	16.9	34.1	20.5	15.4	—	—	16.1
Hawaii	3,506	17.1	28.2	19.2	11.8	27.1	16.5	9.9	16.2	29.9	15.4	—	13.5	—	16.4
Idaho	2,513	18.1	28.5	16.7	8.8	30.3	16.1	6.9	14.7	32.2	18.0	—	—	—	17.8
Illinois	2,581	21.4	33.0	22.6	15.4	34.8	23.7	10.3	19.3	34.0	21.2	22.5	11.9	—	24.2
Indiana	2,380	27.6	45.6	26.6	17.4	41.1	30.1	10.7	22.7	54.9	25.5	32.5	—	—	52.7
Iowa	3,244	20.9	37.9	20.4	11.5	35.2	20.3	7.4	18.2	47.6	20.4	—	—	—	24.4
Kansas	4,499	19.1	31.5	19.5	10.8	32.6	20.9	8.2	16.6	37.8	18.4	22.3	—	—	22.0
Kentucky	3,325	24.7	41.5	24.8	14.5	37.3	28.3	9.5	21.0	48.2	24.7	28.9	—	—	—
Louisiana	2,889	20.2	26.4	23.0	14.7	26.3	25.3	11.1	16.7	35.5	22.2	16.8	—	—	23.9
Maine	3,367	18.7	33.4	18.2	9.8	29.0	22.0	8.5	17.1	29.8	18.6	—	—	—	—
Maryland	4,941	14.9	23.9	19.5	10.0	25.2	19.6	7.3	13.3	27.0	16.4	15.1	4.3	—	8.9
Massachusetts	10,643	15.7	24.7	17.9	11.8	27.7	20.1	7.6	15.1	29.0	16.3	17.4	6.2	—	11.0
Michigan	4,091	20.1	32.8	20.1	13.0	33.6	20.1	10.2	18.1	37.2	19.4	18.5	—	—	33.2
Minnesota	2,355	18.1	31.8	20.4	9.2	30.2	21.5	9.6	16.2	39.9	17.5	21.5	—	—	—
Mississippi	3,259	22.0	28.0	21.9	13.4	29.6	23.1	11.8	18.9	35.4	23.6	19.1	—	—	24.1
Missouri	2,382	25.5	42.9	24.6	15.3	38.6	24.4	12.9	21.9	47.0	25.8	18.9	—	—	—
Montana	3,308	19.5	34.2	16.2	9.4	31.1	21.8	8.2	15.4	36.6	18.4	—	—	37.3	23.6
Nebraska	8,558	20.1	32.7	21.5	10.6	33.3	20.6	9.7	17.2	39.5	20.2	19.6	—	—	21.2
Nevada	2,339	22.1	32.7	22.6	16.8	26.5	26.5	12.5	20.4	29.4	21.7	16.4	21.2	—	22.6
New Hampshire	3,610	18.3	32.0	23.2	11.1	31.8	22.8	8.7	15.7	41.1	18.4	—	1.5	—	—
New Jersey	6,002	16.1	21.2	20.9	11.4	25.1	20.1	8.5	15.3	21.6	17.4	17.7	9.8	—	12.7
New Mexico	2,987	20.5	29.5	17.6	14.2	28.6	22.8	10.4	17.5	32.3	21.0	—	—	11.7	21.9
New York	3,796	17.7	24.5	22.7	10.5	28.5	21.3	8.8	15.9	30.8	18.7	20.1	14.1	—	14.1
North Carolina	7,331	22.0	32.4	21.2	13.8	33.5	21.3	10.1	19.5	33.9	22.8	21.0	22.0	40.3	16.3
North Dakota	2,765	20.4	31.5	19.8	14.3	29.0	25.0	8.8	17.9	39.7	19.2	—	—	53.1	—
Ohio	5,991	20.9	38.1	22.0	9.9	35.7	22.3	7.0	17.7	45.3	20.0	22.7	8.9	—	41.0
Oklahoma	3,398	25.0	37.3	25.1	12.8	34.5	28.2	10.8	21.9	38.0	23.7	28.1	—	31.2	24.4
Oregon	2,218	16.3	23.4	17.1	9.3	29.0	16.3	6.0	13.6	30.6	16.1	—	—	—	11.6
Pennsylvania	5,892	22.8	37.9	24.9	13.6	32.8	25.3	10.9	21.3	34.0	22.4	22.6	22.5	—	24.5
Rhode Island	2,317	18.0	25.5	24.3	11.4	28.7	23.8	8.1	16.2	31.2	18.6	19.7	—	—	11.3
South Carolina	4,388	19.9	28.7	19.8	14.7	29.9	19.6	10.7	16.7	36.2	21.5	15.9	—	—	16.6
South Dakota	3,627	18.7	28.2	17.1	13.6	27.8	20.0	9.9	16.7	33.7	18.0	—	—	37.0	—
Tennessee	1,990	20.7	34.1	17.3	11.8	31.6	18.5	9.9	18.1	35.6	22.8	17.3	—	—	—
Texas	4,767	20.1	26.1	21.4	12.6	28.9	21.3	10.6	16.7	30.5	20.4	19.4	11.9	—	20.6
Utah	2,912	9.8	15.3	13.1	4.4	17.6	8.1	3.4	9.0	13.8	9.5	—	—	—	14.6
Vermont	3,829	16.8	32.7	15.4	7.7	29.2	16.1	8.1	14.6	32.6	16.6	—	—	—	—
Virginia	2,484	16.2	31.9	18.7	9.8	26.0	20.7	8.6	13.6	39.7	16.6	18.1	10.0	—	15.3
Washington	10,576	15.7	26.1	18.7	8.8	28.3	17.4	6.2	13.6	29.9	15.6	20.8	7.1	31.7	16.1
West Virginia	1,794	26.7	40.1	25.4	14.1	37.9	24.2	13.0	22.4	47.6	26.3	—	—	—	—
Wisconsin	3,843	21.5	31.0	20.4	16.8	32.1	22.6	10.9	19.1	43.5	20.7	28.7	—	38.1	37.5
Wyoming	4,295	21.6	39.3	22.4	12.4	33.1	23.9	7.0	17.9	38.9	20.6	—	—	35.7	29.5
**United States**	196,157	19.2	28.9	20.6	11.9	29.3	21.6	9.2	16.8	32.5	19.7	18.7	10.8	27.8	17.9

a Tobacco use is defined as ever having smoked at least 100 cigarettes and currently smoking every day or some days.

b We restricted the prevalence estimates to the categories in which there were 50 or more respondents; blank cells indicate fewer than 50 respondents in this category. Confidence intervals are available from the authors on request.

c The total number of employed respondents in the 2008 BRFSS data stratified by 50 states and Washington, DC (excluding respondents missing smoking data).

**Table 4:** Prevalence of No Influenza Vaccination by State Among Workers Aged 50 to 64 Years, 2008 Behavioral Risk Factor Surveillance System (BRFSS)

State	No. of Respondents^b^	Prevalence of No Influenza Vaccination^a^, %													

		Overall, %	Annual Household Income, $			Educational Attainment			Health Insurance Status		Race/Ethnicity				

			<35,000	35,000-<74,999	≥75,000	High School Graduate or Less	Some College	College Graduate	Any	None	White	African American	Asian/ Hawaiian/ Pacific Islander	American Indian/ Alaska Native	Hispanic
Alabama	1,047	59.9	74.5	57.1	50.6	67.6	62.2	49.7	58.5	73.3	56.3	71.2	—	—	—
Alaska	614	58.2	64.6	61.3	51.2	64.8	58.5	53.2	55.7	73.6	59.6	—	—	46.4	—
Arizona	991	65.0	59.5	66.0	64.5	64.3	69.3	62.2	65.2	63.5	65.4	—	—	—	74.3
Arkansas	1,052	56.2	57.2	56.6	53.5	61.2	54.3	51.8	53.9	72.6	55.6	67.9	—	—	—
California	2,056	63.4	73.9	65.7	57.7	71.6	63.5	58.4	60.3	87.7	58.5	70.2	68.3	—	70.6
Colorado	2,474	53.3	64.6	54.6	49.8	61.1	55.9	48.2	50.9	75.9	52.5	—	—	—	57.4
Connecticut	1,254	54.1	63.6	54.1	51.5	56.3	56.0	52.4	52.9	75.7	52.5	61.9	—	—	70.0
Delaware	769	53.2	66.0	50.2	49.9	67.0	49.6	47.4	52.2	78.4	52.0	59.2	—	—	—
District of Columbia	866	55.5	63.7	58.8	50.6	58.7	61.4	53.0	54.5	68.4	44.7	65.0	—	—	—
Florida	1,911	70.2	77.7	71.1	64.0	78.6	74.4	62.3	67.9	86.2	66.2	74.4	—	—	83.9
Georgia	1,045	62.2	66.5	64.1	58.4	64.5	65.5	58.3	60.7	73.0	60.0	66.4	—	—	—
Hawaii	1,480	53.9	58.4	53.8	52.0	59.2	56.8	49.9	52.6	87.5	58.6	—	45.9	—	50.9
Idaho	1,022	63.8	72.3	68.4	52.8	75.8	63.5	53.9	60.3	86.7	63.5	—	—	—	—
Illinois	1,000	63.7	78.8	65.1	57.8	69.4	64.9	59.1	63.0	73.3	62.8	69.5	—	—	—
Indiana	986	62.6	75.3	61.3	57.6	66.5	61.2	60.0	60.5	90.2	61.7	65.2	—	—	—
Iowa	1,315	52.3	64.2	51.3	47.6	59.0	51.4	44.9	51.7	59.5	52.0	—	—	—	—
Kansas	1,984	57.0	64.7	57.6	53.0	65.9	59.4	50.1	55.3	81.8	56.6	50.5	—	—	62.3
Kentucky	1,362	56.4	69.4	57.5	49.0	66.4	57.6	46.0	54.1	78.1	56.6	—	—	—	—
Louisiana	1,099	56.4	65.5	53.2	52.5	57.7	61.4	52.1	53.7	72.5	55.4	62.3	—	—	—
Maine	1,535	55.2	64.7	57.4	49.3	61.7	52.0	53.1	52.8	76.3	55.2	—	—	—	—
Maryland	1,991	54.6	61.7	59.3	51.6	67.7	53.4	49.1	53.7	68.2	51.3	61.9	—	—	—
Massachusetts	4,210	55.4	66.5	57.2	51.5	60.8	60.5	50.9	55.0	66.3	55.1	65.9	—	—	51.5
Michigan	1,735	60.9	72.1	61.5	56.1	68.9	64.1	53.6	59.6	75.5	59.5	69.8	—	—	—
Minnesota	995	50.4	64.5	51.3	44.4	57.6	51.7	45.4	48.4	88.0	50.0	—	—	—	—
Mississippi	1,360	63.2	71.6	63.1	55.8	68.5	62.0	58.1	60.2	80.5	59.4	70.6	—	—	—
Missouri	1,012	55.8	67.0	56.6	50.7	67.0	56.0	46.4	53.9	76.9	54.2	73.6	—	—	—
Montana	1,550	61.5	71.0	62.3	52.0	71.9	60.2	55.3	58.4	78.6	61.3	—	—	60.7	—
Nebraska	3,794	49.7	59.3	52.5	43.3	57.7	50.4	42.4	47.9	69.0	50.0	—	—	—	58.5
Nevada	922	71.4	83.3	74.7	65.7	82.0	70.7	64.2	69.3	87.8	67.4	—	—	—	84.3
New Hampshire	1,571	52.8	57.8	56.4	50.4	62.9	55.3	47.0	51.2	72.0	52.6	—	—	—	—
New Jersey	2,396	60.7	66.8	66.0	56.7	67.1	61.7	56.3	59.4	74.2	60.2	64.7	—	—	62.3
New Mexico	1,265	62.0	68.5	60.4	57.9	70.2	59.5	58.4	59.4	77.4	60.6	—	—	49.8	65.9
New York	1,614	58.1	69.1	59.5	54.2	65.9	61.1	51.7	55.5	83.3	56.4	70.9	—	—	59.9
North Carolina	2,906	55.0	65.3	55.7	48.6	60.5	55.0	49.6	52.6	74.5	52.9	61.0	—	69.9	60.4
North Dakota	1,202	56.0	55.7	58.0	54.8	62.2	55.5	51.3	54.7	69.2	55.7	—	—	—	—
Ohio	2,749	58.8	61.7	59.9	55.6	64.6	64.2	50.4	57.1	78.4	58.2	61.9	—	—	—
Oklahoma	1,361	49.9	63.6	46.3	43.7	58.6	48.9	41.8	46.5	70.5	50.5	49.4	—	42.7	—
Oregon	1,031	57.9	65.7	62.3	49.9	63.8	59.0	53.3	56.1	75.6	57.3	—	—	—	—
Pennsylvania	2,462	59.2	69.6	57.4	58.0	65.1	60.5	52.3	57.8	77.3	59.1	50.5	—	—	—
Rhode Island	1,008	50.4	54.8	50.5	49.7	55.7	55.1	45.5	49.3	65.7	49.8	—	—	—	—
South Carolina	1,845	59.5	68.8	62.4	51.5	70.5	63.0	47.9	57.5	75.9	58.0	63.7	—	—	—
South Dakota	1,636	47.1	54.0	46.1	43.9	51.6	48.9	41.5	44.6	74.6	47.0	—	—	46.6	—
Tennessee	851	60.7	66.2	59.9	52.5	68.2	60.0	51.9	59.1	71.9	57.6	69.3	—	—	—
Texas	1,883	58.6	66.5	61.8	51.5	67.8	59.9	50.3	56.5	67.8	54.7	71.6	—	—	64.5
Utah	1,004	51.2	67.2	53.0	45.5	58.5	56.1	43.1	49.1	79.4	50.9	—	—	—	—
Vermont	1,759	56.2	62.4	57.3	53.0	63.6	56.3	51.9	54.3	77.8	55.7	—	—	—	—
Virginia	1,043	54.5	49.0	54.7	53.6	59.7	52.4	52.5	53.4	68.7	54.9	67.2	—	—	—
Washington	4,829	56.8	66.8	60.2	51.6	67.2	58.5	51.0	55.0	80.1	57.0	—	43.2	55.3	55.9
West Virginia	754	54.3	68.1	52.5	50.1	64.8	48.6	45.6	52.0	73.2	53.4	—	—	—	—
Wisconsin	1,575	57.2	62.8	59.3	51.9	63.7	58.8	49.9	55.4	76.2	56.7	—	—	—	—
Wyoming	1,896	56.9	64.3	59.1	52.7	63.5	59.0	49.3	54.5	75.7	56.8	—	—	—	54.0
**United States**	82,071	59.3	68.6	60.5	54.4	66.3	61.0	53.3	57.4	77.1	57.4	66.8	60.3	56.3	67.1

a We restricted the prevalence estimates to the categories in which there were 50 or more respondents; blank cells indicate fewer than 50 respondents in this category. Confidence intervals are available from the authors on request.

b The total number of employed respondents in the 2008 BRFSS data stratified by 50 states and Washington DC (excluding respondents missing influenza vaccination data).
